# Assessment of Minimal Residual Disease in Ewing Sarcoma

**DOI:** 10.1155/2012/780129

**Published:** 2012-03-12

**Authors:** Lars M. Wagner, Teresa A. Smolarek, Janos Sumegi, Daniel Marmer

**Affiliations:** ^1^Division of Pediatric Oncology, Cincinnati Children's Hospital Medical Center, University of Cincinnati College of Medicine, 3333 Burnet Avenue, Cincinnati, OH 45229, USA; ^2^Division of Human Genetics, Cincinnati Children's Hospital Medical Center, University of Cincinnati College of Medicine, 3333 Burnet Avenue, Cincinnati, OH 45229, USA; ^3^Division of Bone Marrow Transplantation, Cincinnati Children's Hospital Medical Center, University of Cincinnati College of Medicine, 3333 Burnet Avenue, Cincinnati, OH 45229, USA

## Abstract

Advances in molecular pathology now allow for identification of rare tumor cells in cancer patients. Identification of this minimal residual disease is particularly relevant for Ewing sarcoma, given the potential for recurrence even after complete remission is achieved. Using RT-PCR to detect specific tumor-associated fusion transcripts, otherwise occult tumor cells are found in blood or bone marrow in 20–30% of Ewing sarcoma patients, and their presence is associated with inferior outcomes. Although RT-PCR has excellent sensitivity and specificity for identifying tumor cells, technical challenges may limit its widespread applicability. The use of flow cytometry to identify tumor-specific antigens is a recently described method that may circumvent these difficulties. In this manuscript, we compare the advantages and drawbacks of these approaches, present data on a third method using fluorescent in situ hybridization, and discuss issues affecting the further development of these strategies.

## 1. Introduction

Ewing sarcoma (ES) is the second most common bone tumor in children and young adults. The majority of ES patients have tumors localized to one bone, with no metastases identified using the conventional assessment techniques of imaging and pathologic examination of the bone marrow. However, treatment with aggressive surgical removal alone cures only 10% of these patients [1], while the remaining patients develop fatal metastatic disease presumably arising from otherwise undetected tumor cells in tissues such as lungs or bone marrow that were present at least transiently in the blood. Chemotherapy is used to eradicate this minimal residual disease (MRD), although clinicians have no routine method for knowing the extent of MRD which remains in any given patient. If a reliable, sensitive, and widely applicable assay could be developed for MRD detection in ES, there would be several obvious applications. First, since the finding of MRD in patients with localized tumors is associated with worse outcome [2], these patients could be identified early on for more appropriate high-risk therapies. Second, MRD testing could be used to assess patients for ongoing response to chemotherapy [3], particularly after surgical removal of tumor when imaging no longer can be used to monitor changes in tumor characteristics. Third, identification of the return of low levels of disease may allow for early identification of relapsing patients who have already completed therapy [4–6]. Finally, MRD assessment may be beneficial with clinical decision making in patients with equivocal imaging findings, such as nonspecific lung nodules identified on computed tomography scans [7], as it may support the diagnosis of relapsed disease.

Therefore, given all these potential benefits, investigators have tried for the past two decades to identify methods that are not just sensitive and specific, but widely applicable and feasible in multicenter trials. Ewing sarcoma is well suited for such investigation, given its characteristic genetic and immunophenotypic features which allow for distinction of tumor cells from normal hematopoietic cells. In this manuscript, we review the successes and challenges of the two most common methodologies employed for MRD detection in ES. In addition, we present preliminary data using a third molecular assay and describe an ongoing clinical trial designed to directly compare these assessment strategies.

## 2. RT-PCR for MRD Detection

The majority of studies to assess MRD in ES patients have focused on the use of reverse transcriptase-polymerase chain reaction (RT-PCR) to identify tumor-specific fusion transcripts. This method is based on the fact that approximately 85% of ES tumors are characterized by the *EWS-FLI1* translocation [8]. Tumors not containing *FLI1* translocations usually have other partners for *EWS,* including *ERG*, *ETV1*, *E1AF*, and *FEV*. RT-PCR is attractive for use in MRD detection because of its excellent specificity as well as its sensitivity, determined in spiking experiments to be one tumor cell in one million mononuclear blood cells [9]. In the largest RT-PCR study to date, 20% of 107 ES patients who were considered to have localized tumors using conventional assessments did indeed have evidence of micrometastatic disease in the peripheral blood [2]. Interestingly, 19% of such patients also had MRD identified in the bone marrow, although there was incomplete overlap between those with MRD at either site. Importantly, patients with MRD at either site had worse event-free survival compared to other patients with localized disease, thus showing the potential utility of MRD assessment as a prognostic indicator in a prospective study. Multiple other smaller RT-PCR trials have confirmed that up to one-fourth of newly diagnosed patients with apparently localized tumors have MRD detectable by RT-PCR in blood or marrow [2, 5, 9–14]. Other important applications demonstrated with RT-PCR testing include the ability to assess the efficacy of induction chemotherapy regimens [9] as well as novel purging techniques for peripheral blood stem cell grafts [15]. In addition, several studies have demonstrated that MRD testing can identify relapse in patients before it is clinically apparent by conventional imaging studies [5, 6, 16].  Table 1 summarizes some of the important RT-PCR studies done to date, and these trials provide confirmation of the potential clinical relevance of MRD testing in this disease.

Limitations of RT-PCR include the potential for contamination causing false positive results as well as degradation of mRNA resulting in false negative results [3]. The latter may be particularly important for multicenter trials in which same-day testing is not available. Another potential drawback of RT-PCR is that prior knowledge of the patient's specific translocation is needed so that the appropriate primer sets can be used (*EWS-FLI1* versus *EWS-ERG* versus other). Without this knowledge, interpretation of negative test results is difficult. Historically, RT-PCR had often been performed on initial tumor biopsies as a confirmatory test to support the diagnosis of ES. However, because of technical limitations such as sample size, tissue viability, or absence of frozen tissue, RT-PCR of biopsy material is not always feasible. For example, in the largest multicenter study of RT-PCR to detect MRD in ES, only 117 (68%) of 172 patients had adequate tissue allowing identification of translocations by RT-PCR [2]. It is likely that this number may continue to decrease given the now widespread use of fluorescent *in situ* hybridization (FISH) probes to identify translocations involving *EWS *[17], which can readily be done on paraffin-embedded tissue.

One way to circumvent the requirement for knowledge of the specific translocation partners is to instead assess for individual genes universally expressed in tumor cells but not hematopoietic cells. Cheung et al. used gene expression array data to identify 3 such genes meeting these criteria: *STEAP1, CCND1, *and* NKX2-2 *[18]. The expression of at least one of these 3 genes in histologically negative bone marrow samples from 35 Ewing sarcoma patients was associated with progression-free and overall survival. Additional follow-up studies using this approach have not yet been reported.

## 3. Flow Cytometry for MRD Detection

Another strategy that obviates the need to know the specific translocation is to use multiparameter flow cytometry to identify surface expression of tumor cell antigens. For example, CD99 is universally present on ES tumor cells, and immunostaining for this protein has routinely been used to confirm the diagnosis of ES in primary tumor samples [19]. However, since CD99 is also expressed on some blood cells as well, negative selection for the leukocyte common antigen CD45 is used to exclude hematopoietic cells. Further, to reduce the low level background positivity seen in normal blood and marrow samples, an additional sequential gating strategy is used with a viability dye to remove dead cells, CD14 to exclude monocytes, and CD34 to remove early hematopoietic progenitors which may not yet express CD45. This strategy was used in the first published report of flow cytometry for MRD detection in ES by Dubois et al. [20]. They showed that residual ES cells from two different cell lines can reliably be detected in spiking experiments of peripheral blood and bone marrow at the level of 1 tumor cell in 500,000 or 1 tumor cell in 10,000 mononuclear cells, respectively.

**Figure 1:**
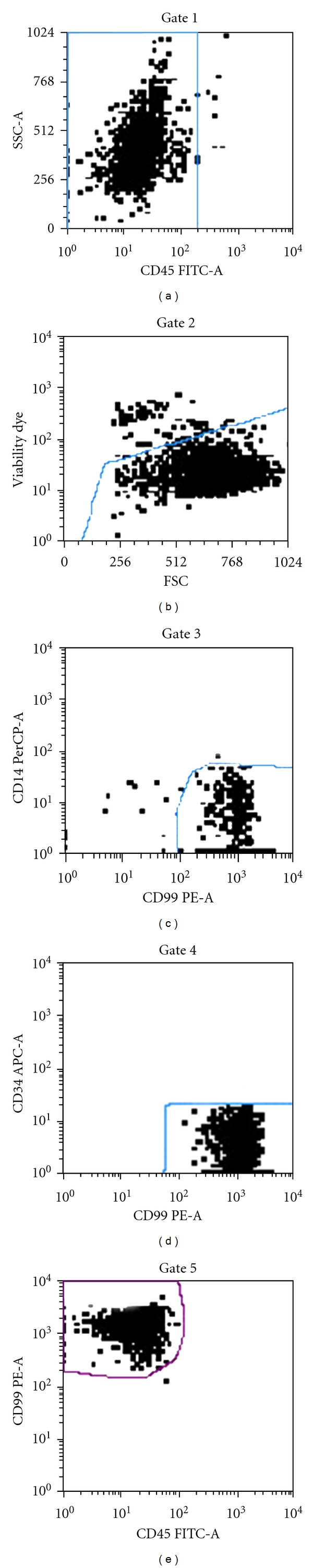
Sequential gating to identify Ewing sarcoma cells. Cultured A673 cells undergo sequential gating to identify Ewing sarcoma cells. Mononuclear cells are separated from blood or marrow by density gradient centrifugation, stained with monoclonal antibodies (CD99 PE, CD45 FITC, CD14 PerCP, CD34 APC), exposed to anti-PE magnetic microbeads to enrich CD99+ cells using MACS technology (Miltenyi Biotec, Cologne, Germany), and then analyzed by flow cytometry. Analysis is performed using sequential gating strategy (gates 1–5) to purify the CD99 bright positive CD45 negative tumor cells as shown in this example of A673 Ewing sarcoma culture cells.

**Figure 2:**
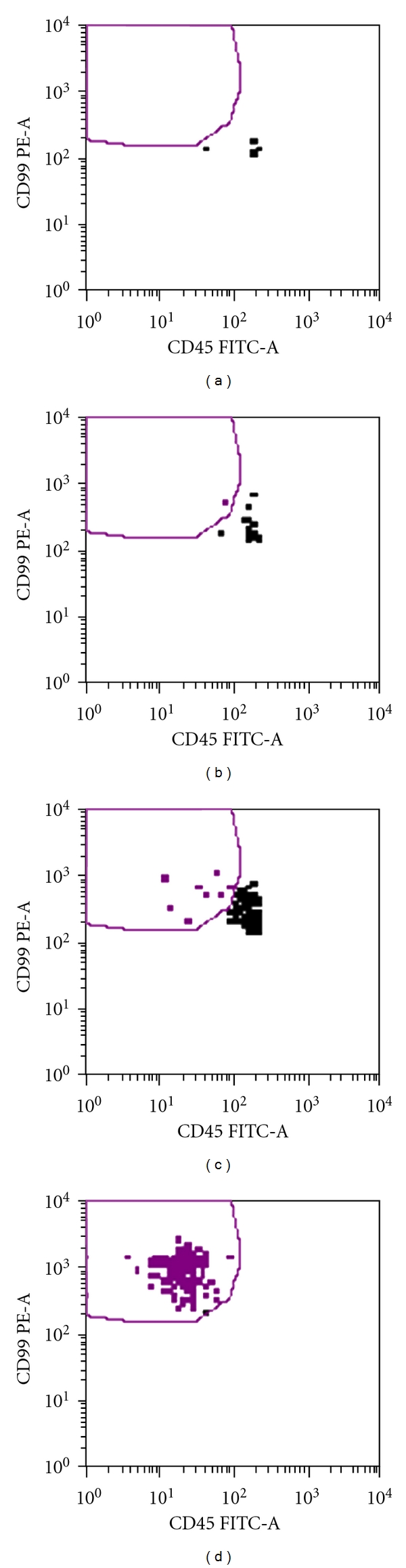
Example of how enrichment can improve the identification of cultures A673 Ewing sarcoma cells mixed with peripheral blood mononuclear cells: (a) No tumor cells are identified in a healthy volunteer blood sample analysis not containing tumor cells (negative control). (b) Conventional flow cytometry without enrichment identifies equivocal findings of a single event (red dot) in a sample containing one A673 Ewing sarcoma cell per 1 × 10^6^ pbmc. (c) In contrast, use of enrichment technique allows for confident identification of tumor cells (e.g., cluster of 5 or more events) that are CD99+/CD45- in a sample containing one A673 cell per 1 × 10^6^ pbmc. (d) Positive control containing only A673 tumor cells.

We have instituted a clinical trial which uses the sequential gating strategy employed by Dubois and shown in Figure 1. In addition, we have modified the assay by incorporating magnetic microbeads to enrich the tumor cell concentration in the residual sample. Variations on this enrichment approach have been described previously [21] and can increase the confidence at which low numbers of tumor cells can be identified.  Figure 2 demonstrates how identification can be improved through enrichment of CD99+ cells. Notably, when enrichment techniques are used, sensitivity in spiking experiments is similar or better to that achieved with RT-PCR, with identification of tumor cells at the range of 1 in one million or more blood mononuclear cells.

Ash and colleagues have recently reported an alternative flow cytometry method which identifies tumor cells expressing both CD99 and CD90 but which are negative for a hematopoietic panel including CD45, CD3, CD14, CD16, and CD19. CD90 is a cell surface protein expressed on some hematopoietic and nonhematopoietic stem cells as well as Ewing sarcoma cells [22]. They assessed previously frozen archival bone marrow samples from 46 patients, including 35 with localized tumors, as well as 10 control samples from patients without malignancy. While the control samples remained negative, CD99+/CD90+ cells were identified in all tested cell lines and patient samples. The range of tumor burden identified in the patient samples was 0.001–0.4%, and the reported sensitivity of the assay using spiking experiments was 0.001% (one tumor cell in 100,000 mononuclear cells). Tumor cells identified by this method were then tested for expression of CD56, which is an isoform of neural cell adhesion molecule (NCAM) found in natural killer cells and neuroectodermal derivatives, including Ewing sarcoma [23]. Sixty percent of the 45 diagnostic samples had high levels of CD56 expression (defined as present in >22% of tumor cells), and this identified a group with greater risk of recurrence. In fact, in this study high CD56 expression in CD99+/CD90+ cells was determined to be an independent prognostic marker with an 11-fold risk of relapse. Although these results should be confirmed in additional studies, they underscore that identification of molecular prognostic markers may be another potential application of flow cytometry.

The fact that prior knowledge of a patient's specific translocation status is not required may make flow cytometry a relevant MRD assessment tool for all ES patients. In addition, the assay is rapid and less labor-intensive than RT-PCR, uses commercially available antibodies, and is well suited for overnight delivery and analysis at a central laboratory. For example, in children with acute lymphoblastic leukemia, flow cytometry performed in a central reference laboratory to assess response to induction therapy has been a feasible and reliable prognostic marker in multi-institutional studies [24] and has become part of the risk assessment strategy on Children's Oncology Group trials.

## 4. FISH for MRD Detection

Another potential method to assess MRD is the use of a FISH break-apart probe to identify translocations involving the *EWS* gene. This method is now commonly used as an adjunct to pathological diagnosis of ES in primary tumor samples. Although many FISH probes do not identify the partner gene for *EWS*, recent studies suggest the specific translocation partner does not hold prognostic significance for patients treated with contemporary therapy [8], and so knowledge of which gene fuses with *EWS* may no longer be relevant for the routine care of ES patients. FISH can also be readily performed on peripheral blood or bone marrow samples and has been used to monitor MRD in leukemia patients [25]. However, there are no previous reports to our knowledge of using FISH in ES for this purpose.

**Figure 3:**
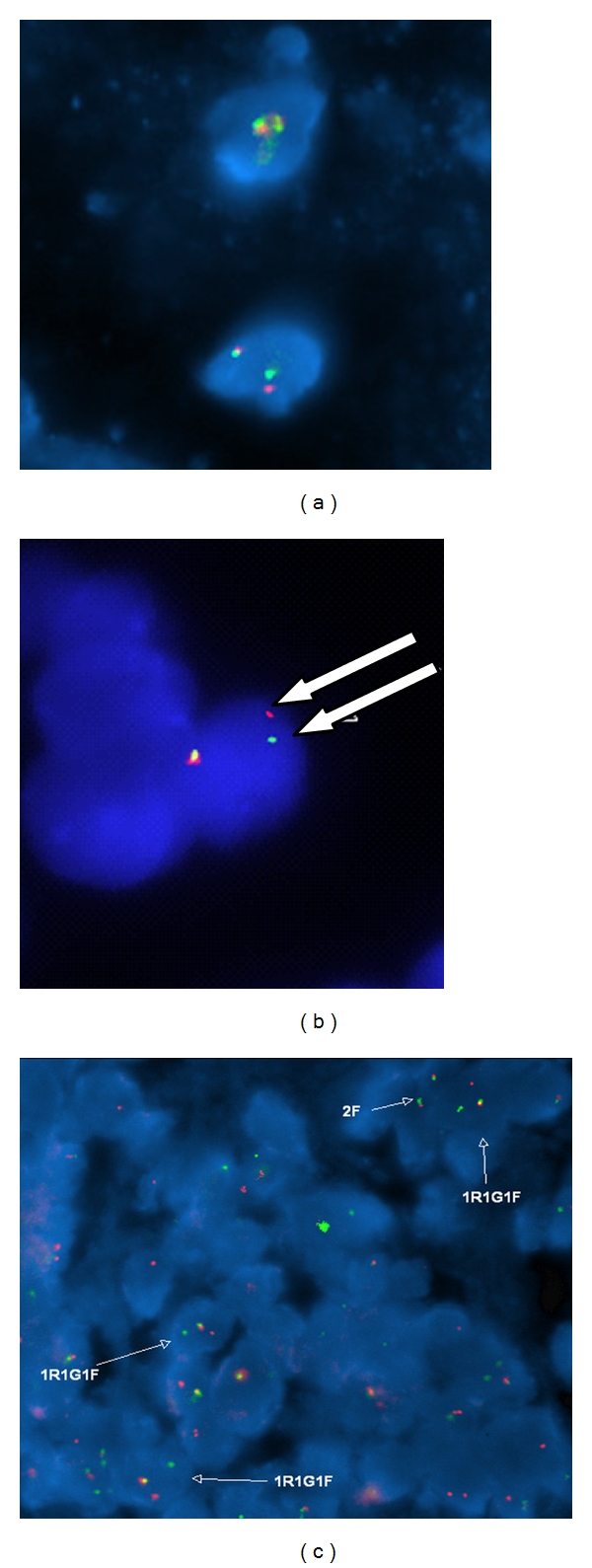
Use of FISH to detect Ewing sarcoma cells. (a) Fluorescence in situ hybridization (FISH) signal pattern for normal cells using the *EWSR1* break-apart probe (Abbott Molecular) showing two fusion signals (red and green signal next to each other with little to no gap in between the signals), which is the normal pattern. (b) FISH signal pattern from normal cells with an occasional false-positive signal pattern (separation of one of the red and green signal pairs with a gap between the two signals wider than the size of one signal alone; see arrows) for *EWSR1* rearrangement. (c) In a sample containing Ewing sarcoma, there is widespread separation of one signal pair in multiple tumor cells (labeled as 1R1G1F), compared to normal cells (labeled as 2F).

In our institution, up to 500 cells are routinely counted when testing for minimal residual disease, which by definition limits the sensitivity to this number. However, potential advantages of FISH testing include the ability to easily test archived samples and the clear visual conformation of the characteristic tumor-specific change in the *EWS* gene. However, even this can sometimes be difficult, depending on the probe being used. A false positive interpretation may occur due to DNA decondensation, which may cause the probes to be sufficiently separated to mimic a true break-apart event. This finding can generally be recognized by an expert cytogeneticist and must be carefully considered when interpreting positive samples.  Figure 3 demonstrates findings seen in normal cells, cancer cells, and in cells deemed to be false positives due to this stretching artifact.

At our institution, we have conducted FISH testing on bone marrow aspirate samples from a limited number of ES patients for the past 5 years, using the Vysis *EWSR1* dual-color break apart probe gene localized to chromosome 22q12 (Abbott Molecular, Abbott Park, IL). The tests were obtained for clinical reasons at the discretion of the treating physician and so were not ordered in any systematic fashion. In fact, testing was not necessarily done on consecutive patients, or even on all samples from an individual patient. Generally, bone marrow samples were pooled together from both sides for a single analysis. FISH testing was performed on 21 bone marrow aspirates from 9 ES patients with either newly diagnosed or relapsed disease who were undergoing evaluations for routine clinical care at Cincinnati Children's Hospital. Of these 21 pooled samples, 14 were negative for tumor by both standard pathology assessment and FISH. In 6 samples from 3 patients, likely tumor cells were identified by FISH alone, with no tumor identified on conventional pathology evaluation. In those patients, the percentage of cells reported with possible *EWSR1 *rearrangement ranged from 0.2% to 7% (median 2.5%) of 200–500 tested mononuclear cells. One patient sample had unequivocal tumor cells identified by morphology on the bone marrow aspirate and biopsy but was negative by FISH. The reason for this false negative remains unclear, as FISH readily showed the characteristic *EWSR1* break apart in the primary bone tumor, as well in a subsequent bone marrow sample done after induction chemotherapy, in which a low level of residual tumor cells was identified despite conventional morphology showing bone marrow remission. We conclude from this limited preliminary data that FISH analysis may detect tumor at low levels not appreciated by conventional morphology in 29% of samples, although one false negative test did occur.

Because the ideal method of MRD assessment in ES is unknown at this time, we are currently performing a trial which prospectively compares RT-PCR versus flow cytometry versus FISH in blood and marrow samples collected from ES patients. Results will be compared between methods as well as with bone marrow pathology reports and imaging studies to correlate the utility of MRD testing with other standard methods of disease assessment. Multiple institutions are participating, which will allow us to assess the feasibility of shipping samples overnight and testing the following day in a central laboratory.

## 5. Additional Issues regarding MRD Assessment

There are several issues which must be worked out for MRD assessment to have broad utility in ES. First, it is unclear which site (blood or bone marrow) will ultimately provide the greatest clinical relevance. In patients with extensive tumor burden, assessment of either site is likely to yield the same result, although these patients will benefit the least from MRD testing because their disease is already clinically apparent. For patients diagnosed with initially localized disease, the impact of minimal bone marrow involvement on outcome has been inconsistent in smaller studies [5, 14]. However, results were more convincing in the largest trial to date [2], which reported a decrease in 2-year disease-free survival from 80% versus 53% when bone marrow MRD testing was positive (*P* = 0.043). It is possible that this may reflect that the impact on outcome is only apparent when a sufficiently large number of patients are tested. Another factor potentially leading to variable results is that bone marrow involvement in ES is more heterogeneous than that in leukemia, and it is common for morphology assessments of disease to differ between sides, and between the aspirates and core biopsies. This was evident in our institutional experience using FISH, in which one patient had aspirates from each side analyzed separately, with disparate results (3% versus 7%).

There is somewhat less data available regarding analysis of circulating tumor cells in ES. As with bone marrow a convincing effect on survival being related to circulating tumor cells at diagnosis is seen in larger [2] but not some smaller studies [5, 6]. Collection of blood samples is far less cumbersome for patients than bone marrow, and is well suited for long-term monitoring either during or after completion of therapy. In fact, the latter approach may be particularly relevant, as several patients have been reported to have circulating tumor cells prior to clinically apparent relapse [5, 6, 16]. In one of the larger studies, 10 of 11 patients with recurrence had tumor cells identified in blood or bone marrow by RT-PCR prior to overt relapse, with a median time lag of 4.5 months (range 1–24 months) [5]. In our current trial, we are performing peripheral blood MRD evaluations any time patients undergo imaging assessments (at diagnosis, on therapy, or after therapy), while bone marrow testing is only performed when marrow samples would be routinely obtained for clinical purposes.

Quantification of RT-PCR results has not been generally reported, with the exception of Merino et al., who used real-time quantitative RT-PCR to estimate the effectiveness of a bone marrow purging method [15]. It is possible that this approach would provide standardization of methodology and consistency in determining exactly what constitutes a positive test result. Similar standardization attempts would be helpful for flow cytometry, given the difficulties in interpreting results when there are only one or two events in the gated field.

Another question is whether cells identified by these methods are truly cancer cells, as each assay has the potential for false positives. Although RT-PCR detects pathognomonic *EWS* changes not found in hematopoietic cells, contamination during RNA collection and testing may occur. For FISH, changes in the *EWS *gene during decondensation of DNA can cause an occasional cell to appear as if there may be a true rearrangement, as discussed earlier and noted in Figure 2. For flow cytometry, despite the use of a panel of markers to exclude hematopoietic cells, there is always the possibility of illegitimate transcription of these hematopoietic markers in tumor cells. In fact, in the most recent report by Ash et al. [22], flow cytometry was reported to identify tumor cells in all 35 diagnostic bone marrow samples from patients with localized disease, and this incidence of 100% is in sharp contrast to all previous reports estimating the incidence of marrow micrometastases to be 20–30% in this patient population. Because the sensitivity of their assay is within the same range of that reported with RT-PCR, the question is raised whether all of these cells were indeed tumor cells. Efforts to reduce the potential for false positive results should continue.

Other methodologic issues include the specific protocols regarding how samples are collected and in what volume. Using a large volume (10 mL or perhaps more) may be ideal for collecting blood samples, particularly in patients who are on therapy and who may have treatment-related reductions in the number of circulating mononuclear cells. However, more may not necessarily be better with bone marrow collections, as demonstrated in a recent report by Helgestad et al. [26]. They showed that the density of nucleated cells in the bone marrow of leukemia patients is markedly reduced with larger volume aspirates, due to potential dilution with peripheral blood during the collection. In fact, this dilution effect from larger aspirations resulted in several samples being interpreted as negative (below limits of sensitivity by flow cytometry), despite clearly containing >0.1% tumor cells in the first small volume sample withdrawn. Further, bilateral bone marrow aspirations are routinely performed for ES patients, due to the typically patchy tumor involvement. Most studies do not specify whether both sides are pooled together or analyzed separately. Attention to standardization of collection procedures will help improve interpretation of test results.

Finally, it remains unclear which assay has the greatest utility. Because of the success of flow cytometry for MRD assessment in leukemia, the readiness of commercially available antibodies, and the encouraging results noted so far in preliminary studies, it is likely that there will be further exploration of flow cytometry for MRD detection in ES. Results from ongoing trials which directly compare these methodologies will hopefully provide input on which assay to study in larger prospective clinical trials.

## 6. Summary

Detection of MRD in blood or bone marrow is best established for patients with childhood leukemia, where flow cytometry to assess response to therapy is now a standard part of risk assessment [24]. In adult carcinomas, FDA-approved methods like the CellSearch assay identify circulating tumor cells through positive enrichment using epithelial cell-specific Ep-CAM antibodies followed by image analysis [27] and are now widely employed. Among pediatric solid tumors, there has been considerable work in MRD detection in neuroblastoma (reviewed in [28]), which like ES is characterized by disease recurrence following complete remission in a substantial subset of patients. ES appears particularly well suited for MRD detection due to tumor-specific translocations that facilitate RT-PCR and FISH detection as well as expression of tumor-specific cell surface proteins like CD99 that facilitate detection by flow cytometry. Studies using RT-PCR have demonstrated that otherwise occult tumor cells can indeed be identified at initial diagnosis in the blood and/or marrow of approximately one-fifth of ES patients with otherwise localized disease and that such patients generally have inferior outcome. Smaller studies have shown that return of MRD as detected in the blood or marrow often precedes clinically apparent relapse and that MRD assessment can be used to follow response to chemotherapy regimens. Although effective therapeutic interventions for these findings may not yet be available in some cases, the results to date support the contention that clinically meaningful information can be obtained from assessing MRD in ES patients, and that further study is indicated.

While the aforementioned studies have used RT-PCR, flow cytometry offers a commercially available, less labor-intensive approach with similar sensitivity that may be more widely applicable, given that detection does not require prior knowledge of the particular chromosomal translocation. Also, this method may be less susceptible to degradation of sample integrity if overnight shipping to a central laboratory is required. However, further validation in additional studies is required, and standardization of sample collection, testing methods, and reporting of results will be critical. Trials are currently underway which will compare these modalities to each other, and to compare MRD test results with imaging studies and overall outcome to further define the overall utility and clinical relevance of MRD assessment in this disease.

## Figures and Tables

**Table 1:** Summary of Key Studies Using RT-PCR for MRD Detection in Ewing Sarcoma.

Author [Reference]	N (pts)	Key Findings
Peter [9]	36	31% had MRD in either blood or marrow at diagnosis at diagnosis
Pfeiderer [13]	16	38% had MRD in BM, while only 6% had CTC at diagnosis
West [12]	28	25% of newly diagnosed localized pts had MRD in blood or marrow, compared to 50% with relapsed/metastatic disease
Fagnou [11]	67	26% of patients had MRD in blood at diagnosis, but this was not correlated with clinical features or outcome.33% of patients had BM MRD, with worse outcome
De Alava [6]	28	MRD in blood and/or marrow developed prior to clinical progression
Zoubek [14]	35	7/23 (30%) had BM MRD at diagnosis, but this did not predict relapse
Thomson [3]	9	Survival was correlated with speed of clearance of MRD in blood and BM
Sumerauer [10]	22	MRD in marrow found in 31% of localized and 50% of metastatic pts
Merino [15]	12	Quantitative RT-PCR can be used to measure efficacy of stem cell purging methods
Schleiermacher [2]	172	MRD in blood or BM at diagnosis is associated with worse survival in patients with otherwise localized disease
Avigad [5]	26	43% of pts had marrow MRD at diagnosisMRD developed prior to clinical recurrence in 10 of 11 patients
Yaniv [16]	11	Tumor cells frequently contaminate stem cell harvests, and are associated with relapse after transplantation.Relapse is preceded by MRD in BM and/or blood

MRD, minimal residual disease; BM, bone marrow.
